# A Validation Study on the Frequency and Natural History of Miscarriages Using the Spanish Primary Care Database BIFAP

**DOI:** 10.3390/healthcare9050596

**Published:** 2021-05-18

**Authors:** Sara Sanchez Ortiz, Consuelo Huerta, Ana Llorente-García, Paloma Ortega, Paloma Astasio, Lucía Cea-Soriano

**Affiliations:** 1 Department of Preventive Medicine and Public Health, Faculty of Medicine, Complutense University of Madrid, 28040 Madrid, Spain; sarsan06@ucm.es (S.S.O.); mahuer05@ucm.es (C.H.); pomolina@ucm.es (P.O.); pastasio@ucm.es (P.A.); 2 BIFAP, Division of Pharmacoepidemiology and Pharmacovigilance, Spanish Agency for Medicines and Medical Devices (AEMPS), 28040 Madrid, Spain; allorente_externo@aemps.es

**Keywords:** validation studies, pregnancy, primary care databases, miscarriages, risk factors

## Abstract

(1) Background: There is a major gap of knowledge towards the natural history of miscarriages in electronic medical records. We aimed to calculate the frequency of miscarriages using data from BIFAP database. (2) Methods: We identified all pregnancy losses and carried out a multistep validation exercise. Potential cases with positive predictive values (PPV) of miscarriage confirmation <85% or those confirming other pregnancy loss were excluded. Kaplan–Meier figures and incidence rates (IRs) of miscarriage with 95% confidence intervals (CIs) expressed by 1000 person-weeks were calculated. Stratifying analysis by age, specific high-risk groups, and drug exposure within the pre-pregnancy period were performed restricted to women with recording last menstrual period (LMP). (3) Results: Women with confirmed miscarriage (*N* = 18,070), tended to be older, with higher frequency of comorbidities and drug utilization. Restricting to women with LPM recorded, IR of miscarriage was 10.89 (CI 95% 10.68–11.10) per 1000 women-weeks, with a median follow-up of 10 weeks (IQR: 8–12). The IR according to age was: 2.71 (CI 95% 2.59–2.84) in those aged <30 years compared to 9.11 (CI 95% 8.55–9.70) in women aged ≥40 years. Advanced maternal age (Hazard Ratio (HR, 95% confidence interval) CI 95%: 3.34 (3.08–3.62)), use of antihypertensives (1.49 (1.21–1.84), and use of drugs classified as D or X during pregnancy (1.17 (1.07–1.29)) showed to be positive predictors associated with increased risk of miscarriages. (4) Conclusion: BIFAP database can be used to identify women suffering from miscarriages, which will serve to further study risk factors associated with miscarriages with special attention to drug utilization.

## 1. Introduction

Miscarriage accounts for approximately 15–20% of all recognized pregnancies [1]. Maternal age has experienced a global delay on Western countries [2,3,4,5]. In Spain, mean age at delivery has risen from 25.2 years in 1975 to 30.7 years in 2016. The proportion of deliveries corresponding to women aged over 35 years of age was 39% and 8% for women aged over 40 years old. This later childbearing has been observed worldwide, in both high- and low-income countries [6,7,8,9,10].

In terms of causality, miscarriages are considered of multifactorial nature. Among plausible factors, those can be classified as unmodifiable or modifiable ones. Examples of unmodifiable factors include chromosomal abnormalities of the fetus, representing around 50% of the miscarriages or inherited susceptibility [11]. Among modifiable factors, the most common ones include life style factors such as smoking, alcohol intake, obesity but also psychological stress. It has been estimated how preventing and attenuating the exposure of these factors to lower levels might avoid up to 25% of all cases [1,12,13]. Advanced maternal age might carry a higher risk of maternal comorbidity such as diabetes, hypertension, and obstetric complications [14,15,16,17].

Although miscarriages are a frequent fatal outcome of pregnancy, it is difficult to establish the specific time window occurrence and therefore to estimate its frequency rate. Among its challenges and bias on the identification, the following scenarios should be considered: (i) some episodes would go unnoticed if occurring during the first few weeks of gestational age, (ii) not all countries register miscarriages but stillbirths (from 28 weeks onwards), lacking a complete data source to be analyzed distorting the true rate, and (iii) some episodes could be recorded as unspecified abortions rather than specify miscarriages. Although electronical medical records have become the gold standard for pharmacoepidemiological and epidemiological studies, several challenges are needed to keep in mind when studying pregnancy outcomes. First, to accurately identify the timing of pregnancy as the beginning or end of pregnancy are often missing and not systematically recorded, and secondly, another issue is to identify and validate the outcome of pregnancy. For the latter, validation studies are warranted on distinguish across pregnancies losses such as miscarriages, terminations of pregnancies, or unspecified abortions. There have been prior studies validating these outcomes using secondary data such as registries [18], emergency department [19] obtaining high positive predictive values. However, no prior studies have validated these outcomes using the Spanish primary care database BIFAP.

Based on the current gap in the identification of miscarriages and its onset using electronical medical records, this study aimed to quantify the frequency of miscarriages as well as to determine the specific time window for its occurrence using data from BIFAP database. Identifying risk factors and selected conditions would help to build existing data for further studies which aimed to monitor the drug safety during pregnancy.

## 2. Materials and Methods

### 2.1. Source of Data

We used data from Spanish database Base de Datos para la Investigación Farmacoepidemiológica en Atención Primaria, Database for Pharmacoepidemiological Research in Primary Care (BIFAP). BIFAP is a computerized medical longitudinal population-based database of anonymized electronic medical records of primary care practitioners and pediatricians (PCP) from nine participating Autonomous Regions (out of 17) in Spain. BIFAP includes information of 6857 primary care physicians and pediatricians, including: demographic factors, consultation visits, referrals, hospital admissions, laboratory test results, diagnostic procedures, diagnoses, and prescriptions. BIFAP’s age and sex distribution are comparable to the Spanish population, covering 8.6% of the total Spanish population at the time this study was performed [20,21]. Clinical encoding of diagnoses and symptoms, are included using two coding systems: International Classification of Primary Care—Second Edition (ICPC-2) and ICD-9. The ICPC is the coding system for eight out of nine participant Autonomous Regions, and its granularity is limited as compared with ICD-9 [22,23]. Prescriptions issued by the PCP are automatically recorded; prescriptions from specialists as well as those used during hospitalizations may not be fully captured. In addition, from 2011 onwards, e-prescription has progressively been implemented in primary care centers, therefore dispensation is also available. Prescriptions are entered using the ATC classification [24]. The information is then harmonized into BIFAP common data model, details on the BIFAP database have been described previously [25]. The study protocol was approved by the BIFAP Scientific Committee (Reference #11/2016).

### 2.2. Source Population

The source population included all women of childbearing age (15–49 years) with at least one-year registration with their PCP between January 2002 to December 2015. The study cohort was restricted to all women with an entry compatible with pregnancy during the study period. For the current study, we only included one pregnancy per woman, the first pregnancy identified during the follow-up. Within this subsample, we applied several operational definitions to determine the beginning and end of gestation during the study period. We used an adaptation from a valid algorithm designed by the authors and applied in other database with similar characteristics [26]. Details of the identification of a cohort of pregnancies, as well as the determination and imputation of gestational age have been described elsewhere [27]. The cohort encompassed a total of 155,419 women. Out of them, 77.5% of pregnancies were completed (*N* = 120,469), 21.5% resulted in pregnancies losses (which included miscarriages, TOPs, and unspecified abortions) (*N* = 33,342), 0.8% were ectopic (*N* = 1285), and 0.2% were stillbirth (*N* = 323). A total of 101,307 (65.2%) women had recorded the LMP date on their profiles while for the remaining 54,112 (34.8%) the LMP date was imputed. Among women with LMP date recorded, we estimated the time interval (gestational age) according with the end of pregnancy. Women with delivery/postpartum or stillbirth when gestational age was < 155 days (22 weeks) or >320 days were excluded; women with a pregnancy loss or ectopic pregnancy and a gestational age <28 days or >154 days (22 weeks) were excluded. The median days from LMP date to abortion date/ectopic pregnancy date were considered as gold standard, obtaining 74 and 52 days, respectively. Finally, when LMP date was not recorded, we imputed it. For women with a delivery or stillbirth, the LMP date was imputed by subtracting 280 days. For women with an entry of abortion, we imputed the LMP date by subtracting 74 days to the recorded entry date and 52 days when there was a code of ectopic pregnancy [27].

### 2.3. Separating Miscarriages from All Pregnancy Losses Validation Steps

**Figure 1:**
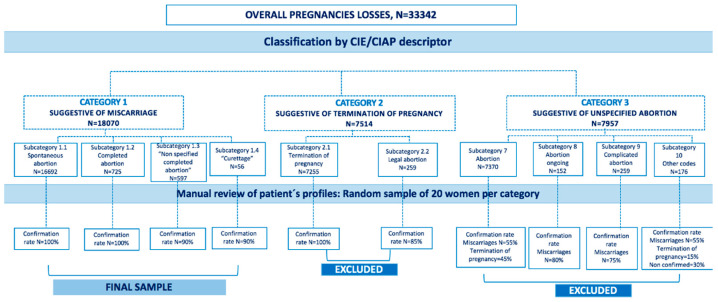
Flow chart of study design and identification of pregnancy losses.

In BIFAP, there are several codes to record pregnancy loss, some of them are suggestive of miscarriages, other indicate termination of pregnancies (TOP) and others are unspecified and do not allow to differentiate a spontaneous abortion (i.e., miscarriage) from a TOP. There have not been prior studies focusing on abortions in BIFAP that might help to validate several codes suggesting different fatal events in pregnancy. Out of the 33,342 identified pregnancy losses using these codes, we subdivided women in three main categories according to the code and descriptor used to entry the episode (Figure 1): category 1: referring to potential miscarriages outcomes; category 2: referring to potential termination of pregnancy outcomes; and category 3: referring to potential unspecified abortion outcomes. Thus, per each category, we further created several granulated subcategories according to the specificity and detailed information provided in their descriptors to classify the outcome of interest. Among women with a suggestive indicator for miscarriage (category 1), we subdivided them into several subcategories: subcategory 1.1, encompassing all women with a code which contains the term “spontaneous abortion” (*N* = 16,692); subcategory 1.2, including all women with a code suggestive of “completed abortion” (*N* = 725); subcategory 1.3, encompassing all women with a code suggestive of “non-specified completed abortion” (*N* = 597); subcategory 1.4, extending to all women with a code suggestive of “undergoing a curettage process”, (*N* = 56). Among women with a suggestive indicator for termination of pregnancy (category 2), we subdivided them into the following subcategories: subcategory 2.1, encompassing all women with an entry code of “termination of pregnancy” (*N* = 7255); and subcategory 2.2, including 259 women with an entry code of “legal abortion“. Finally, among women with a suggestive indicator for unspecified abortion (category 3), we subdivided them into the following subcategories: subcategory 3.1 including all women with a code suggestive of “abortion” (*N* = 7370); subcategory 3.2 encompassing all women with a code suggestive of “abortion on going” (*N* = 152); subcategory 3.3 encompassing all women with a code suggestive of “complicated abortion“ (*N* = 60); and subcategory 3.4 extending to all remaining women (*N* = 176).

### 2.4. Validation of Categories of Pregnancy Losses: Miscarriage, Termination of Pregnancy, and Unspecified Abortion

For each subcategory presented above, we selected a random sample of medical records of each subcategory and manually reviewed them. Since our goal was to study the frequency of miscarriages as well as to study principal risk factors, all categories of codes with a positive predictive value (PPV) below 85% were excluded from the study. Figure 1 shows the confirmation rates. Among women classified as potential miscarriages (i.e., classified under category 1) subcategories 1.1 and 1.2 had a PPV of 100% and 90% for subcategories 1.3 and 1.4. For women with an entry suggestive of termination of pregnancy (subcategories 2.1 and 2.2) the confirmation rate was 100% and therefore all women were considered as having a termination of pregnancy. Finally, among women with a suggestive entry of unspecified abortion, we obtained the following results: 55% for subcategory 3.1 (i.e., abortion), 80% for subcategory 3.2, 75% for subcategory 3.3, and 55% for subcategory 3.4, all them were excluded from the pool of miscarriages (i.e., PPV < 85%). Our final sample of women suffering from miscarriages was 18,070 (54% of all pregnancy losses).

### 2.5. Cohort Analysis

To study the incidence rate of miscarriages in BIFAP, we used the whole cohort of pregnancies (27) and followed them up until the occurrence of the following end points, whichever came first:

- Pregnancy loss (i.e., miscarriage, TOP, and unspecified abortions);
- 154 days (22 weeks) that was the upper limit of the gestational age to consider a pregnancy loss;
- Death.

### 2.6. Ascertainment of Exposure and Covariates

For baseline characteristics, we considered all the information available in the database any time prior to the LMP date, prioritizing the information closer to LMP. Variables abstracted included life style factors such as smoking, demographic characteristics such as women’s age and body mass index (calculated from recorded height and weight; weight in kg/(height in meters2), most prevalent illnesses, prescriptions, and health care utilization indicators.

### 2.7. Ascertainment of Drug Exposure

Exposure to medication was defined as the presence of at least one prescription restricting to the pre-pregnancy period (defined as the 90 days before the LMP date). We analyzed the frequency of prescription of the most frequently prescribed drugs (with at least a prevalence of 0.5%); and selected drugs classified by the FDA as category “D and X”.

In order to identify high risk profiles for miscarriages, we grouped women according to several metabolic and/or psychiatric conditions ascertained any time prior LMP date. First, we created a grouped called “metabolic antecedents” defined as the presence of any of the following conditions: obesity, diabetes, hypercholesterolemia or other alteration related with cholesterol, altered arterial blood pressure, and alterations in glycosylated hemoglobin levels in women. Second, neuropsychiatric antecedents defined as having recorded in their profiles either epilepsy, ADHD, anxiety, depression, or eating disorders.

### 2.8. Analysis

We described the characteristics of women with confirmed miscarriages compared with the rest of pregnancy cohort using frequency counts and percentages for categorical variables, and means with standard deviation for continuous variables. Incidence rates of miscarriage with 95% confidence intervals (CIs) expressed by 1000 person-weeks were calculated for overall and stratified by age, specific high-risk groups, and drug exposure within the pre-pregnancy period. Cumulative incidence of miscarriages with 95% confidence intervals (CIs) were also calculated. Kaplan–Meier survival functions with log rank test were performed to compare the survival distributions across groups. We conducted a sensitivity analysis restricted to women who had recorded the LMP date in order to accuracy measured the exact gestational age (time interval from LMP date to miscarriage date). Adjusted Hazard Ratios of miscarriages and 95% confidence intervals (CIs) were calculated and adjusted by age, number of PCP visits, and year of pregnancy restricted to women with recorded LMP date. STATA version 12.0 was used for all analyses.

## 3. Results

### 3.1. Baseline Characteristics

Our pregnancy cohort encompassed a total of 155,419 women, out of them 33,342 had a pregnancy loss (21.5%). After complete validation of all potential cases, there was a total of 18,070 (11.6%) women with a miscarriage. Table 1 shows the frequency of baseline characteristics and comorbidities according to outcome of interest (i.e., miscarriages vs. remaining pregnant cohort) and also by recorded gestational age (having LMP date recorded in the database vs. imputed). Overall, women with miscarriage tended to be older compared with the remaining cohort (i.e., 31.5% were aged 35–39 years compared with 24.7% in the remaining cohort and 12.1% vs. 4.9% were aged 40 years and more (*p-*value < 0.001). They also tended to have slightly higher frequency of common conditions such as depression (+1.7%), anxiety (+1.5%), and hypothyroidism (+13%) (*p* < 0.001). They presented slightly higher frequency of drug prescriptions within the pre-pregnancy period. Overall, excluding mineral and vitamins, a total of 49.8% of women with miscarriage received at least one prescription compared with 46.3% of the remaining pregnant cohort and 6.0% (*p* < 0.001) and 4.5% when restricting to categories D and X of the FDA classification (*p* ≤ 0.001), respectively.

When focusing on women with a confirmed miscarriage, there were no substantial differences across those with LMP date recorded and those with imputed LMP date in terms of distribution of demographics, comorbidities of drug utilization (Table 1).

### 3.2. Overall Incidence of Miscarriage

Overall, cumulative incidence of miscarriage was 12% (8% among subsample of women with LMP date recorded and 21% among those imputed), corresponding incidence rate was 5.98 (CI 95% 5.90–6.07) per 1000 women-weeks (10.89 (CI 95%: 10.68–11.10) and 3.77 (CI 95%: 3.68–3.85), respectively.

### 3.3. Specific Groups Restricted to Women with LMP Date Recorded

**Figure 2:**
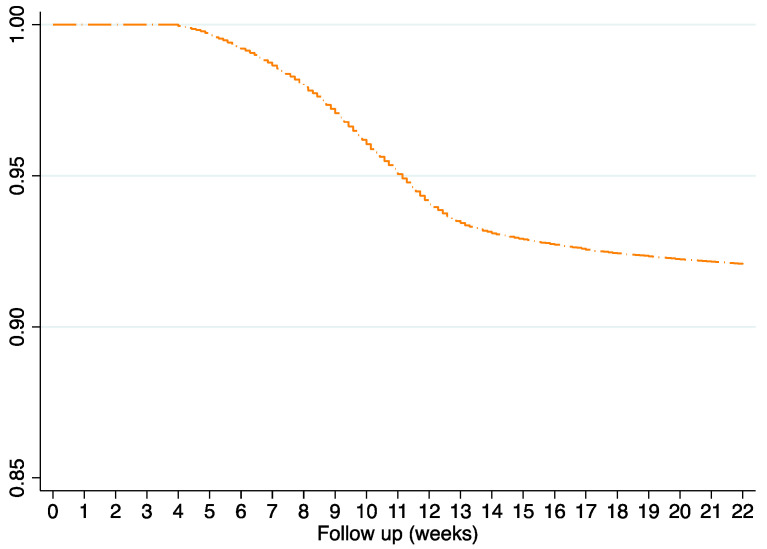
Kaplan–Meier survival estimate showing time to miscarriage onset restricted to women with LMP date recorded.

The lines that follow describe the results restricting to women with LMP date recorded (*N* = 101,307). Supplementary Table S1 shows the cumulative incidence, incidence rate, median weeks, and log rank tests. The overall median follow-up was 10 weeks (IQR 8–12) (Figure 2).

**Figure 3:**
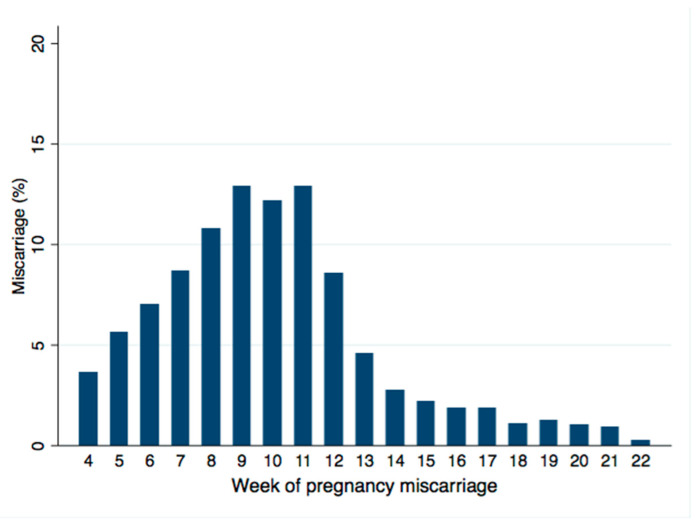
Bar graph showing the distribution of weeks of miscarriage occurrence restricted to women with LMP date recorded.

The highest time window risk was observed from weeks 7 to 12, accounting for 66% of miscarriages (Figure 3).

**Figure 4:**
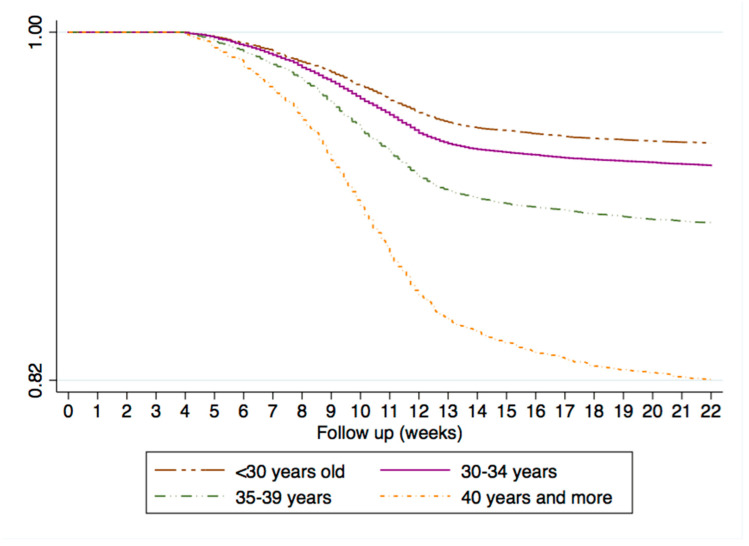
Kaplan–Meier survival estimate showing time to miscarriage onset according to age and restricted to women with LMP date recorded.

Figure 4 shows the Kaplan–Meier survival function of miscarriages by age. There was in increased risk of miscarriage according to age (log rank tests: *p* < 0.001). The incidence of miscarriage per 1000 women-weeks according to age was: 2.71 (95% CI: 2.59–2.84) in those aged <30 years compared to 9.11 (95% CI: 8.54–9.70) in women aged 40 years or above, log rank tests *p* < 0.001 (Supplementary Table S1). At week 10, 60% of women aged <30 years old suffered the miscarriage compared to 68% of women aged 40 years and above (data not shown).

When evaluating specific conditions, women with metabolic antecedents had a higher incidence of miscarriage (IRs 4.25 (95% CI: 4.00–4.51), likewise for psychiatric antecedents (IRs 4.80 (CI 95% 3.98–5.78)) (Supplemental Figure S1 and Supplemental Table S1). Women receiving at least one drug during pre-pregnancy period presented a higher incidence of miscarriage (IRs 4.05 (CI 95%:3.94–4.18)) compared with women not receiving any drug (3.52 (95% CI: 3.41–3.63) long rank test: *p* < 0.001. The same trend was found among women receiving at least one drug labeled as D and X according to FDA classification (5.01 (CI 95%: 4.58–5.49) and 3.71 (CI 95%: 3.62–3.79), log rank test *p* < 0.001, respectively) (Supplemental Figure S2 and Supplementary Table S1).

### 3.4. Predictors for Miscarriages

This analysis was also restricted to women with LMP date recorded. The multivariate Cox regression model was adjusted by age, number of PCP visits, and year of LMP date. Age showed to be a strong positive predictor associated with miscarriages (Table 2), corresponding adjusted HRs of miscarriages were as follows: HR of 1.21 (95% CI: 1.14–1.28) for women aged of 30–34 years, 1.76 (95% CI: 1.65–1.87) aged 35–39, and 3.34 (95% CI: 3.09–3.62) aged ≥40 years. There was a positive trend of miscarriage associated to number of PCP visits (i.e., HR 1.23 (95% CI: 1.15–1.31) for 2–4 visits compared to 1.45 (95% CI: 1.35–1.56) for ≥10 visits. We did not find a clear trend according to year of LMP date.

In terms of comorbidities, prior history of cardiopathy was associated with an HR of 3.58 (95% CI 1.49–8.60), hence this result was based on small numbers and conclusions should take care with caution. Some conditions related with the nervous central system tended to be associated with a higher risk although not them all reached the statistical significance: ADHD 1.61 (95% CI 0.80–3.22); migraine 1.08 (95% CI 0.99–1.18); anxiety 1.06 (95% CI 1.00–1.12); and depression 1.12 (95% CI 1.03–1.21). Having at least one prescription of antihypertensives was associated with an HR of 1.49 (95% CI 1.21–1.84); and SSRIs 1.15 (95% CI 0.99–1.34), benzodiazepines 1.13 (95% CI 1.02–1.25). Prescription drugs included in the D and X category carried an adjusted HR 1.17 (95% CI 1.07–1.29).

## 4. Discussion

The present study describes the epidemiology of miscarriages in routine clinical practice in Spain using data from BIFAP database, which is multiregional database with a total of 9 Autonomous Regions participating which might be representative of the Spanish population in terms of age and sex [20]. Our study encompassed a total of 155,419 pregnant women identified between 2002 and 2015 in BIFAP, applying a previous and novel algorithm [26]. A validation study based on identification of specific descriptors together with a manual review of the patient’s profiles was performed to identify and confirm women with a miscarriage as a fatal result of pregnancy. For codes in which its terminology implicitly suggests the event of interest such as “miscarriages/spontaneous abortion/” or “termination of pregnancies/legal abortion” the PPV ranged from 90 to 100%. However, unspecified groups such as “abortions” or complicated abortion presented a PPV of miscarriages <85% and therefore these women could not be considered.

Within our cohort of pregnancies, the proportion of women with miscarriage was 12% with an incidence rate of 5.98 per 1000 women-weeks. Prior observational studies also reported similar rates of miscarriages, ranging from 10 to 20% [28,29,30,31,32]. These results support the validity of BIFAP database to further evaluate the underlying risk factors for its onset. The incidence of miscarriage was strongly associated to the increased of maternal age, being almost 4-fold times higher among women aged 40 years and above compared with women aged <30 years. These results are in line with previous studies, where women who were aged 45 years and above had a percentage of miscarriage of 56.9%. [21,22,23]. Based on these findings, we additionally explored the secular trends of maternal age in our cohort taking extreme years of the study period: 2002–2005 vs. 2013–2015. While the frequency of pregnant woman older than 40 years of age was 5.4% during the first part of the study, this proportion increased up to 8.22% by 2013–2015. As a counterpart, the proportion of women aged <30 years decreased from 33.5% vs. 26.2%, respectively. These trends observed have been reported previously [12,33].

With the increase of maternal age, it is more likely that women present complications during pregnancy [34,35]. Advanced maternal age has been also associated with other pregnancy outcomes such as preterm delivery [36,37,38], low birth weight, perinatal death, and cesarean section. Preestablished diabetes and hypertension are one of the most common comorbidities within pregnant women which might impact not only on the conditions for pregnant woman but also on the offspring. Indeed, previous studies have also observed how hypertension might increase risk of miscarriage [39], as well as diabetes [40] and cardiovascular diseases [41,42]. As a mechanism on this increased risk, it has been postulated a placental vascular pathology which might be enhanced with increasing maternal age [43]. Our study shows how the proportion of metabolic conditions has risen from 1.9% by 2002/2013 up to 6% in the last years of the study period. Prescription drugs during pre-pregnancy have also increased during the study period; however, drugs categorized as X and D remained constant (5.0% vs. 4.7%). We found how several central nervous system diseases such as depression and anxiety as well as its treatment (benzodiazepines and/or SSRIs) slightly increased the risk of miscarriages. This association is still under debate [44]; thus, further studies are warranted in order to evaluate the underlying cause for miscarriages and other fatal pregnancy outcomes.

### Strengths and Limitations

The strengths of this study include the use of a large sample of pregnancies (*N* = 155,419) among a multiregional representative sample of Spanish women of childbearing age. This cohort is the result of applying a valid algorithm in a primary care database, BIFAP [26,27]. We found that there were no differences of distribution of life style factors and other covariates among pregnant women with LMP date compared with imputed LMP date which reinforce the validity of the pregnant cohort. We might have missed some miscarriage cases resulting from women who go to private clinics to monitor their pregnancy or abortion, many miscarriages that are managed at home and go unreported and some not even reported to a clinician. These scenarios can also explain why some women had no assigned LMP date, gestational age, or date of the event. Thus, the algorithm used was not validated by sending questionnaires to the PCP in order to validate both the gestational age and type of outcome of pregnancy, however, the distribution of women who had miscarried (77.51% completed vs. 21.45% pregnancy losses) are in line with previous studies using similar data sources [26,45,46,47]. In addition, the frequency of miscarriages obtained in women with recorded LMP and women with imputed LMP date was very similar, which supports the truthfulness of our study. In terms of information of prescription drugs, BIFAP contains information based on prescriptions or dispensing medications (the latter progressively since 2011), this means that the prescription does not reflect the real use of drugs and there may be an overestimation in taking of those. On the other hand, there could be an underestimation in taking those drugs that were dispensed without a prescription, or with a private prescription.

## 5. Conclusions

The findings of the current study show how a primary care database such as BIFAP can be used to identify women suffering from miscarriages. For instance, this validated cohort will allow to deeply evaluate specific risks among subgroup populations such as those suffering from metabolic or central nervous system conditions.

## Figures and Tables

**Table 1:** Baseline characteristics of women according to miscarriage status and LMP date entry.

Baseline Characteristics	Women with Miscarriage				Remaining Pregnant Women			
	LMP Date Recorded *N* = 7827		LMP Date Imputed *N* = 10,243		LMP Date Recorded *N* = 93,480		LMP Date Imputed *N* = 43,869	
	N	%	N	%	N	%	N	%
Age								
<30 years	1731	22.1	2161	21.1	29,051	31.1	12,845	29.3
30–34 years	2685	34.3	3265	31.9	36,744	39.3	15,655	35.7
35–39 years	2462	31.5	3204	31.3	23,072	24.7	11,986	27.3
≥40 years	949	12.1	1613	15.7	4613	4.9	3383	7.7
Obesity	612	7.8	735	7.2	6540	7.0	2549	5.8
Smoke	683	8.7	982	9.6	7160	7.7	3267	7.4
Anemia	50	0.6	51	0.5	414	0.4	171	0.4
Anxiety	1472	18.8	1766	17.2	16,132	17.3	6581	15.0
Asthma	398	5.1	525	5.1	4648	5.0	2082	4.7
Depression	745	9.5	978	9.5	7271	7.8	3329	7.6
Diabetes	66	0.8	114	1.1	562	0.6	311	0.7
Epilepsy	48	0.6	65	0.6	549	0.6	289	0.7
Hypercholesterolemia	318	4.1	449	4.4	3516	3.8	1438	3.3
Hypertension	123	1.6	153	1.5	951	1.0	493	1.1
Hypothyroidism	612	7.8	643	6.3	6057	6.5	2248	5.1
Irritable Bowel Syndrome	160	2.0	199	1.9	1719	1.8	712	1.6
Migraine	534	6.8	689	6.7	5770	6.2	2434	5.5
Multiple Sclerosis	10	0.1	12	0.1	153	0.2	64	0.1
Psychiatric antecedents *	110	1.4	151	1.5	1031	1.1	528	1.2
Metabolic antecedents ^φ^	167	2.1	168	1.6	1599	1.7	574	1.3
Acid suppressant	406	5.2	552	5.4	4397	4.7	1890	4.3
Antibiotics	947	12.1	1205	11.8	10,746	11.5	4607	10.5
Antiepileptics	55	0.7	93	0.9	576	0.6	337	0.8
Antihistamines	379	4.8	437	4.3	4242	4.5	1687	3.8
Antihypertensives	89	1.1	121	1.2	518	0.6	327	0.7
Antimigraine drugs	66	0.8	79	0.8	604	0.6	239	0.5
Benzodiazepines	416	5.3	549	5.4	3880	4.2	1909	4.4
Codeine	74	0.9	101	1.0	883	0.9	430	1.0
Folic Acid	744	9.5	624	6.1	8302	8.9	2227	5.1
Oral Corticosteroids	181	2.3	277	2.7	2305	2.5	995	2.3
Respiratory drugs	504	6.4	564	5.5	5649	6.0	2195	5.0
SSRIs	179	2.3	255	2.5	1597	1.7	885	2.0
Thyroid hormone	212	2.7	208	2.0	1798	1.9	610	1.4
All drugs except vit/minerals	3900	49.8	4513	44.1	43,300	46.3	16,800	38.3
D and X category	471	6.0	611	6.0	4210	4.5	2097	4.8

* Psychiatric antecedents included at least one of the following entities: epilepsy, attention deficit hyperactivity disorder (ADHD), anxiety, depression, and eating disorders. ^φ^ metabolic antecedents included at least one of the following entities: presence of obesity, diabetes, hypercholesterolemia or other alteration related with cholesterol, altered arterial blood pressure, and alterations in glycosylated hemoglobin levels in women. Baseline characteristics and comorbidities were ascertained any time before LMP date (identified or estimated) selecting the most recent data. Categories D/X according to FDA classification.

**Table 2:** Hazard Ratio of miscarriages among women with LMP date recorded.

	Women with Miscarriages *N* = 7827		Women w/o Miscarriages *N* = 93,480		HR (95% CI) ^ϕ^
	N	%	N	%	
Age					
<30 years	1731	22.1	29,051	31.1	1 (-)
30–34 years	2685	34.3	36,744	39.3	1.21 (1.14–1.28)
35–39 years	2462	31.5	23,072	24.7	1.76 (1.65–1.87)
>40 years	949	12.1	4613	4.9	3.34 (3.08–3.62)
GP Visits					
0–1	1388	17.7	20,438	21.9	1 (-)
2–4	2312	29.5	27,627	29.6	1.23 (1.15–1.31)
5–9	2478	31.7	28,620	30.6	1.27 (1.19–1.36)
>10	1649	21.1	16,795	18.0	1.45 (1.35–1.56)
Smoking	683	8.7	7160	7.7	1.07 (0.91–1.25)
Anxiety	1472	18.8	16,132	17.3	1.06 (1.00–1.12)
Cardiopathy	5	0.1	15	0.0	3.58 (1.49–8.60)
Depression	745	9.5	7271	7.8	1.12 (1.04–1.21
Epilepsy	48	0.6	549	0.6	1.00 (0.75–1.33)
Hypertension	123	1.6	951	1.0	1.19 (1.00–1.43)
Hypothyroidism	612	7.8	6057	6.5	1.09 (1.00–1.18)
Irritable Bowel Syndrome	160	2.0	1719	1.8	1.04 (0.89–1.22)
Migraine	534	6.8	5770	6.2	1.08 (0.99–1.18)
Metabolic conditions *	1092	14.0	11,557	12.4	1.05 (0.98–1.11)
Psychiatric conditions ^φ^	110	1.4	1031	1.1	0.90 (0.74–1.09)
Antibiotics	947	12.1	10,746	11.5	0.99 (0.92–1.06)
Acid Suppressants	406	5.2	4397	4.7	0.99 (0.89–1.10)
Benzodiazepines	416	5.3	3880	4.2	1.13 (1.02–1.25)
Antihypertensives	89	1.1	518	0.6	1.49 (1.21–1.84)
ARBs	30	0.4	204	0.2	1.21 (0.85–1.74)
ACEI	9	0.1	50	0.1	1.37 (0.71–2.64)
Calcium channel blockers	11	0.1	53	0.1	1.78 (0.99–3.22)
B-Blocking agents	40	0.5	181	0.2	2.07 (1.52–2.83)
Diuretics	19	0.2	96	0.1	1.59 (1.01–2.49)
SSRI	179	2.3	1597	1.7	1.15 (0.99–1.34)
Antimigraine drugs	66	0.8	604	0.6	1.17 (0.92–1.49)
Folic Acid	744	9.5	8302	8.9	1.02 (0.94–1.10)
Antiepileptics	55	0.7	576	0.6	1.02 (0.79–1.22)
Thyroid Hormones	212	2.7	1798	1.9	1.15 (1.01–1.33)
D X category	471	6.0	4210	4.5	1.17 (1.07–1.29)
All drugs (exc vit and min)	3900	49.8	43,302	46.3	1.03 (0.98–1.08)

^ϕ^ HR adjusted by age, year of LMP date, and number of visits.* Metabolic antecedents included at least one of the following entities: presence of obesity, diabetes, hypercholesterolemia or other alteration related with cholesterol, altered arterial blood pressure, and alterations in glycosylated hemoglobin levels in women. ^φ^ Psychiatric antecedents included at least one of the following entities: epilepsy, attention deficit hyperactivity disorder (ADHD), anxiety, depression, and eating disorders. Baseline characteristics and comorbidities were ascertained any time before LMP date (identified or estimated) selecting the most recent data.

## Data Availability

Data will be available upon request.

## Supplementary Materials

The following are available online at https://www.mdpi.com/article/10.3390/healthcare9050596/s1, Figure S1: Kaplan–Meier survival estimate showing time to miscarriage onset according to metabolic antecedents (left figure) and according to psychiatric disorders (right figure) and restricted to women with LMP date recorded. Figure S2. Kaplan–Meier survival estimate showing time to miscarriage onset according to receiving at least one medication (left figure) and according to receiving at least one medicine classified as D or X according to FDA classification and restricted to women with LMP date recorded. Table S1. Cumulative incidence of miscarriage per 1000 women and incidence rate of miscarriage per 1000 women-weeks according to specific risk profile groups.

## References

[1] Poorolajal J., Cheraghi P., Cheraghi Z., Ghahramani M., Doosti Irani A. (2014). Predictors of miscarriage: A matched case-control study. Epidemiol. Health.

[2] Magnus M.C., Wilcox A.J., Morken N.H., Weinberg C.R., Håberg S.E. (2019). Role of maternal age and pregnancy history in risk of miscarriage: Prospective register based study. BMJ.

[3] Cooke A., Mills T.A., Lavender T. (2012). Advanced maternal age: Delayed childbearing is rarely a conscious choice a qualitative study of women’s views and experiences. Int. J. Nurs. Stud..

[4] Londero A.P., Rossetti E., Pittini C., Cagnacci A., Driul L. (2019). Maternal age and the risk of adverse pregnancy outcomes: A retrospective cohort study. BMC Pregnancy Childbirth.

[5] De la Rochebrochard E., Thonneau P. (2002). Paternal age and maternal age are risk factors for miscarriage; results of a multicentre European study. Hum. Reprod..

[6] Radoń-Pokracka M., Adrianowicz B., Płonka M., Danił P., Nowak M., Huras H. (2019). Evaluation of Pregnancy Outcomes at Advanced Maternal Age. Open Access Maced. J. Med. Sci..

[7] Ogawa K., Urayama K.Y., Tanigaki S., Sago H., Sato S., Saito S., Morisaki N. (2017). Association between very advanced maternal age and adverse pregnancy outcomes: A cross sectional Japanese study. BMC Pregnancy Childbirth.

[8] Ben-David A., Glasser S., Schiff E., Zahav A.S., Boyko V., Lerner-Geva L. (2016). Pregnancy and Birth Outcomes Among Primiparae at Very Advanced Maternal Age: At What Price?. Matern. Child Health J..

[9] Almeida N.K.O., Almeida R.M.V.R., Pedreira C.E. (2015). Adverse perinatal outcomes for advanced maternal age: A cross-sectional study of Brazilian births. J. Pediatr..

[10] Wang Y., Tanbo T., Åbyholm T., Henriksen T. (2011). The impact of advanced maternal age and parity on obstetric and perinatal outcomes in singleton gestations. Arch. Gynecol. Obstet..

[11] Woolner A.M.F., Raja E.A., Bhattacharya S., Danielian P., Bhattacharya S. (2020). Inherited susceptibility to miscarriage: A nested case-control study of 31,565 women from an intergenerational cohort. Am. J. Obstet. Gynecol..

[12] Feodor Nilsson S., Andersen P.K., Strandberg-Larsen K., Nybo Andersen A.M. (2014). Risk factors fot miscarriage from a prevention perspective: A nationwide follow up study. BJOG.

[13] Bhattacharya S. (2015). Modifiable risk factors for miscarriage identified. Evid. Based Nurs..

[14] Bu Z., Hu L., Su Y., Guo Y., Zhai J., Sun Y.P. (2020). Factors related to early spontaneous miscarriage during IVF/ICSI treatment: An analysis of 21,485 clinical pregnancies. Reprod. Biomed. Online.

[15] McDonnell R., Hart R.J. (2017). Pregnancy-related outcomes for women with polycystic ovary syndrome. Womens Health.

[16] Combs C.A., Kitzmiller J.L. (1991). Spontaneous abortion and congenital malformations in diabetes. Baillieres Clin. Obstet. Gynaecol..

[17] McGrogan A., Snowball J., de Vries C.S. (2014). Pregnancy losses in women with Type 1 or Type 2 diabetes in the UK: An investigation using primary care records. Diabet. Med..

[18] Lohse S.R., Farkas D.K., Lohse N., Skouby S.O., Nielsen F.E., Lash T.L., Ehrenstein V. (2010). Validation of spontaneous abortion diagnoses in the Danish National Registry of Patients. Clin. Epidemiol..

[19] Quinley K.E., Falck A., Kallan M.J., Datner E.M., Carr B.G., Schreiber C.A. (2015). Validation of ICD-9 Codes for Stable Miscarriage in the Emergency Department. W. J. Emerg. Med..

[20] Maciá-Martínez M.A., Gil M., Huerta C., Martín-Merino E., Álvarez A., Bryant V., Montero D., BIFAP Team (2020). Base de Datos para la Investigación Farmacoepidemiológica en Atención Primaria (BIFAP): A data resource for pharmacoepidemiology in Spain. Pharmacoepidemiol. Drug Saf..

[21] Gobernanza del Acceso a los Datos de BIFAP Agencia Española de Medicamentos y Productos Sanitarios (n.d.). 28 January 2021. http://bifap.aemps.es/docs/Gobernanza_acceso_datos_BIFAP_v1_Junio2017.pdf.

[22] World Organization of Family Doctors (WONCA) International Classification Committee (1998). International Classification of Primary Care (ICPC).

[23] Ministerio de Sanidad, Servicios Sociales e Igualdad, Dirección General de Salud Pública, Calidad e Innovación, Subdirección General de Información Sanitaria e Innovación (n.d.). Edición Electrónica de la CIE-9- MC. Clasificación Internacional de EnferMedades 9a Revisión, Modificación Cínica. 28 January 2021. https://eciemaps.mscbs.gob.es/ecieMaps/browser/index_9_mc.html.

[24] WHO Collaborating Centre for Drug Statistics Methodology (n.d) ATC/DDD Index. 28 January 2021. https://www.whocc.no/atc_ddd_index/.

[25] Gil M., Rodríguez-Miguel A., Montoya-Catalá H., González-González R., Álvarez-Gutiérrez A., Rodríguez-Martín S., García-Rodríguez L.A., de Abajo F.J. (2019). Validation study of colorectal cancer diagnosis in the Spanish primary care database, BIFAP. Pharmacoepidemiol. Drug Saf..

[26] Cea-Soriano L., García Rodríguez L.A., Fernández Cantero O., Hernández-Díaz S. (2013). Challenges of using primary care electronic medical records in the UK to study medications in pregnancy. Pharmacoepidemiol. Drug Saf..

[27] Sanchez Ortiz S., Llorente García A., Astasio P., Huerta C., Cea Soriano L. (2020). An algorithm to identify pregnancies in BIFAP Primary Care database in Spain: Results from a cohort of 155 419 pregnancies. Pharmacoepidemiol. Drug Saf..

[28] Wilcox A.J., Weinberg C.R., O’Connor J.F., Baird D.D., Schlatterer J.P., Canfield R.E., Armstrong E.G., Nisula B.C. (1988). Incidence of early loss of pregnancy. N. Engl. J. Med..

[29] Almeida N.D., Basso O., Abrahamowicz M., Gagnon R., Tamblyn R. (2016). Risk of Miscarriage in Women Receiving Antidepressants in Early Pregnancy, Correcting for Induced Abortions. Epidemiology.

[30] Rossen L.M., Ahrens K.A., Branum A.M. (2018). Trends in Risk of Pregnancy Loss Among US Women, 1990–2011. Paediatr. Perinat. Epidemiol..

[31] Adolfsson A., Larsson P.G. (2006). Cumulative incidence of previous spontaneous abortion in Sweden in 1983-2003: A register study. Acta Obstet. Gynecol. Scand..

[32] Hemminki E., Forssas E. (1999). Epidemiology of miscarriage and its relation to other reproductive events in Finland. Am. J. Obstet. Gynecol..

[33] Marinescu I.P., Foarfă M.C., Pîrlog M.C., Turculean A. (2014). Prenatal depression and stress—Risk factors for placental pathology and spontaneous abortion. Rom. J. Morphol. Embryol..

[34] Oppong S.A., Torto M., Beyuo T. Risk factors and pregnancy outcome in women aged over 40 years at Korle-Bu Teaching Hospital in Accra, Ghana. Int. J. Gynaecol. Obstet..

[35] De Viti D., Malvasi A., Busardò F., Beck R., Zaami S., Marinelli E. (2019). Cardiovascular Outcomes in Advanced Maternal Age Delivering Women. Clinical Review and Medico-Legal Issues. Medicina.

[36] Luke B., Brown M.B. (2007). Elevated risks of pregnancy complications and adverse outcomes with increasing maternal age. Hum. Reprod..

[37] Laopaiboon M., Lumbiganon P., Intarut N., Mori R., Ganchimeg T., Vogel J.P., Souza J.P., Gülmezoglu A. (2014). M WHO Multicountry Survey on Maternal Newborn Health Research Networ. Advanced maternal age and pregnancy outcomes: A multicountry assessment. BJOG.

[38] Dietl A., Cupisti S., Beckmann M.W., Schwab M., Zollner U. (2015). Pregnancy and obstetrical outcomes in women over 40 years of age. Geburtshilfe Frauenheilkd.

[39] Abalos E., Duley L., Steyn D.W., Gialdini C. (2018). Antihypertensive drug therapy for mild to moderate hypertension during pregnancy. Cochrane Database Syst. Rev..

[40] Cea-Soriano L., García-Rodríguez L.A., Brodovicz K.G., Masso Gonzalez E., Bartels D.B., Hernández-Díaz S. (2018). Safety of non-insulin glucose-lowering drugs in pregnant women with pre-gestational diabetes: A cohort study. Diabetes Obes. Metab..

[41] Cuneo B.F., Kaizer A.M., Clur S.A., Swan H., Herberg U., Winbo A., Rydberg A., Haugaa K., Etheridge S., Ackerman M.J. (2020). Mothers with long QT syndrome are at increased risk for fetal death: Findings from a multicenter international study. Am. J. Obstet. Gynecol..

[42] Stryuk R.I., Burns C.A., Filippov M.P., Brytkova Y.V., Borisov I.V., Barkova E.L., Gomova T.A., Kozina E.A., Nagirnyak O.A. (2018). Cardiovascular disease and associated comorbid conditions as determinants of adverse perinatal outcomes in pregnancy—An analysis of the results of the register of pregnant BEREG. Ter. Arkh..

[43] Kelly R., Holzman C., Senagore P., Wang J., Tian Y., Rahbar M.H., Chung H. (2009). Placental vascular pathology findings and pathways to preterm delivery. Am. J. Epidemiol..

[44] Richardson J.L., Martin F., Dunstan H., Greenall A., Stephens S., Yates L.M., Thomas S.H. (2019). Pregnancy outcomes following maternal venlafaxine use: A prospective observational comparative cohort study. Reprod. Toxicol..

[45] Blotière P.O., Weill A., Dalichampt M., Billionnet C., Mezzarobba M., Raguideau F., Dray-Spira R., Zureik M., Coste J., Alla F. (2018). Development of an algorithm to identify pregnancy episodes and related outcomes in health care claims databases: An application to antiepileptic drug use in 4.9 million pregnant women in France. Pharmacoepidemiol. Drug Saf..

[46] Charlton R.A., Cunnington M.C., de Vries C.S., Weil J.G. (2008). Data resources for investigating drug exposure during pregnancy and associated outcomes: The General Practice Research Database (GPRD) as an alternative to pregnancy registries. Drug Saf..

[47] Toh S., Mitchell A.A., Werler M.M., Hernández-Díaz S. (2008). Sensitivity and specificity of computerized algorithms to classify gestational periods in the absence of information on date of conception. Am. J. Epidemiol..

