# Supplementary material for: A Validation Study on the Frequency and Natural History of Miscarriages Using the Spanish Primary Care Database BIFAP
Source: Healthcare (Basel). 2021 May 18;9(5):596. doi: 10.3390/healthcare9050596 (PMC8157258; doi:10.3390/healthcare9050596)
Supplement: Supplementary file 1 [file healthcare-09-00596-s001.zip › healthcare-1202942-supplementary.pdf]

Table S1. Cumulative incidence of miscarriage per 1000 women and incidence rate of miscarriage per 1000 women-weeks according to specific risk profile groups.

Group	Nº of Cases	Median Weeks among Cases (IQR)	Person Time	Cumulative Incidence per 1000 women	Incidence Rate per 1000 Women-Weeks	Log Rank Test
**Restricted to sample with LMP date recorded, *N* = 101,307**						
**All women, *N* = 101,307**	7827	10 (8–12)	2,078,295	18.48 (17.67–19.33)	3.77 (3.68–3.85)	N.A.
Age						<0.001
Age <30 years, *N* =30782	1731	10 (7–12)	638,691	56.23 (53.72–58.86)	2.71 (2.59–2.84)	
Age 30–34 years, *N* = 39,429	2685	10 (7–12)	818,944	68.10 (65.65–70.63)	3.28 (3.16–3.41)	
Age 35–39 years, *N* = 25,534	2462	10 (8–12)	516,447	96.42 (92.86–100.10)	4.78 (4.58–4.96)	
Age 40 years and more, *N* = 5562	949	10 (8–11)	104,213	170.62 (160.96–180.73)	9.11 (8.54–9.70)	
Psychiatric antecedents, *N* = 1141	110	10 (7–12)	22,930	96.41 (80.61–114.91)	4.80 (3.98–5.78)	0.0107
Non-Psychiatric antecedents, *N* = 100,166	7717	10 (8–12	2,055,365	77.04 (75.41–78.71)	3.75 (3.67–3.84)	
Metabolic antecedents, *N* = 12,649	1092	10 (7–11)	257,001	86.33 (81.56–91.35)	4.25 (4.00–4.51)	<0.001
Non-metabolic antecedents, *N* = 88,658	6735	10 (8–12)	1,821,294	75.97 (74.24–77.73)	3.70 (3.61–3.79)	
At least one drug (exc. Vits/minerals), *N* = 47,202	3900	9 (7–12)	962,942	82.62 (80.17–85.14)	4.05 (3.93–4.18)	<0.001
No drugs (exc. Vits/minerals), *N* = 54,105	3927	10 (8–12)	1,115,353	72.58 (70.43–74.80)	3.52 (3.41–3.63)	
At least one drug cat D/X, *N* = 4681	471	10 (8–12)	93,947	100.62 (92.33–109.57)	5.01 (4.58–5.49)	<0.001
No drugs cat D/X, *N* = 96,626	7356	10 (8–12)	1,984,347	76.13 (74.47–77.82)	3.71 (3.62–3.79)	

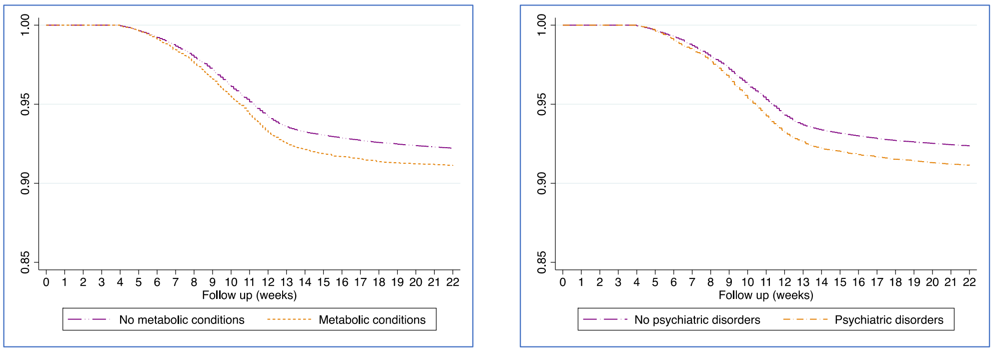

Figure S1. Kaplan–Meier survival estimate showing time to miscarriage onset according to metabolic antecedents (left figure) and according to psychiatric disorders (right figure) and restricted to women with LMP date recorded.

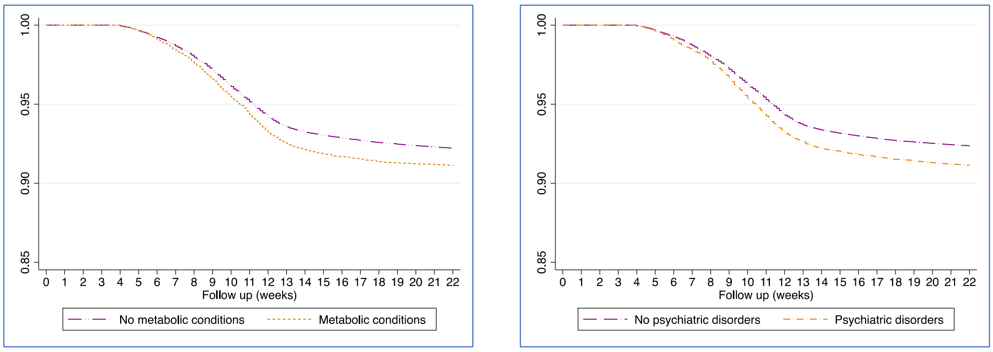

Figure S2. Kaplan–Meier survival estimate showing time to miscarriage onset according to receiving at least one medication (left figure) and according to receiving at least one medicine classified as D or X according to FDA classification and restricted to women with LMP date recorded.
